# Exploring precision therapeutics: computational design of antisense oligonucleotides targeting AXL gene transcripts in multiple sclerosis treatment management

**DOI:** 10.3389/fchem.2025.1548269

**Published:** 2025-02-05

**Authors:** Bhargav Shreevatsa, Abhigna Nagaraj, Chandan Dharmashekar, Anisha Jain, Bhavana Harendra, Siddesh V. Siddalingegowda, Haneen A. Al-Mazroua, Sheikh F. Ahmad, Shashanka K. Prasad, Chandrashekar Srinivasa, Chandan Shivamallu, Shiva Prasad Kollur

**Affiliations:** ^1^ Department of Microbiology, JSS Academy of Higher Education and Research, Mysuru, India; ^2^ Department of Pathology, Microbiology and Immunology, School of Medicine, University of South Carolina, Columbia, SC, United States; ^3^ Department of Biotechnology and Bioinformatics, JSS Academy of Higher Education and Research, Mysuru, India; ^4^ Department of Pharmacology and Toxicology, College of Pharmacy, King Saud University, Riyadh, Saudi Arabia; ^5^ Department of Studies in Biotechnology, Davangere University, Davangere, Karnataka, India; ^6^ School of Physical Sciences, Amrita Vishwa Vidyapeetham, Mysuru, India

**Keywords:** AXL gene, antisense oligonucleotides, multiple sclerosis, neurodegeneration inflammation, therapeutics

## Abstract

Multiple sclerosis (MS) is a chronic autoimmune illness characterized by demyelination, neurodegeneration, and inflammation in the central nervous system. The AXL gene, which codes for a receptor tyrosine kinase, has emerged as a promising therapeutic target due to its involvement in neuroinflammation and oligodendrocyte dysfunction. In the current study, we employed *in silico* techniques to design Antisense Oligonucleotides (ASOs) that selectively target AXL gene transcripts to modulate AXL expression and mitigate MS pathology. Three ASOs, A1, A2, and A3, were designed to specifically target the 5′ untranslated region (5′UTR) and coding region of the AXL gene transcripts. The ASOs were optimized with a focus on stability, binding affinity, and specificity towards AXL mRNA while minimizing off-target effects. To investigate ASO-mRNA interactions and gauge their ability to alter AXL expression, Molecular Docking was performed. Our analyses showed that A1, A2, and A3 had substantial interactions with AXL mRNA, with binding affinities of −9.5 kcal/mol, −10.8 kcal/mol, and −10.6 kcal/mol, respectively. The targeting of AXL gene transcripts through ASOs shows promise in reducing MS symptoms. Precision ASO-based therapies could effectively manage MS by targeting the essential pathways involved in the disease. ASOs provide a highly targeted approach for treating MS and offer a precise therapeutic strategy for this debilitating condition. The study lays the groundwork for future *in vitro* and *in vivo* studies to confirm the therapeutic potential of these ASOs for the treatment of MS.

## 1 Introduction

Multiple sclerosis (MS) is a chronic inflammatory demyelinating autoimmune disorder of central nervous system (CNS) with unknown etiology (Al-Ghezi et al., 2019). It was considered as a rare disease in India in the past, studies from AIIMS Delhi (2015) over time suggest an increased incidence of MS in India. The precise prevalence of MS in India is not well known due to a dearth of epidemiological research, diagnoses, and registries that have been properly undertaken. However, by the current report from World Health Organization (WHO) and Multiple Sclerosis International Federation (MSIF) 2013, the crude prevalence rate of MS in India is about 5–20 per 100,000 which is much higher than the studies reported previously. Globally, MS prevalence has increased dramatically, affecting more than 2.3 million people (Bhatia et al., 2015; Zahoor and Haq, 2017).

**FIGURE 1:**
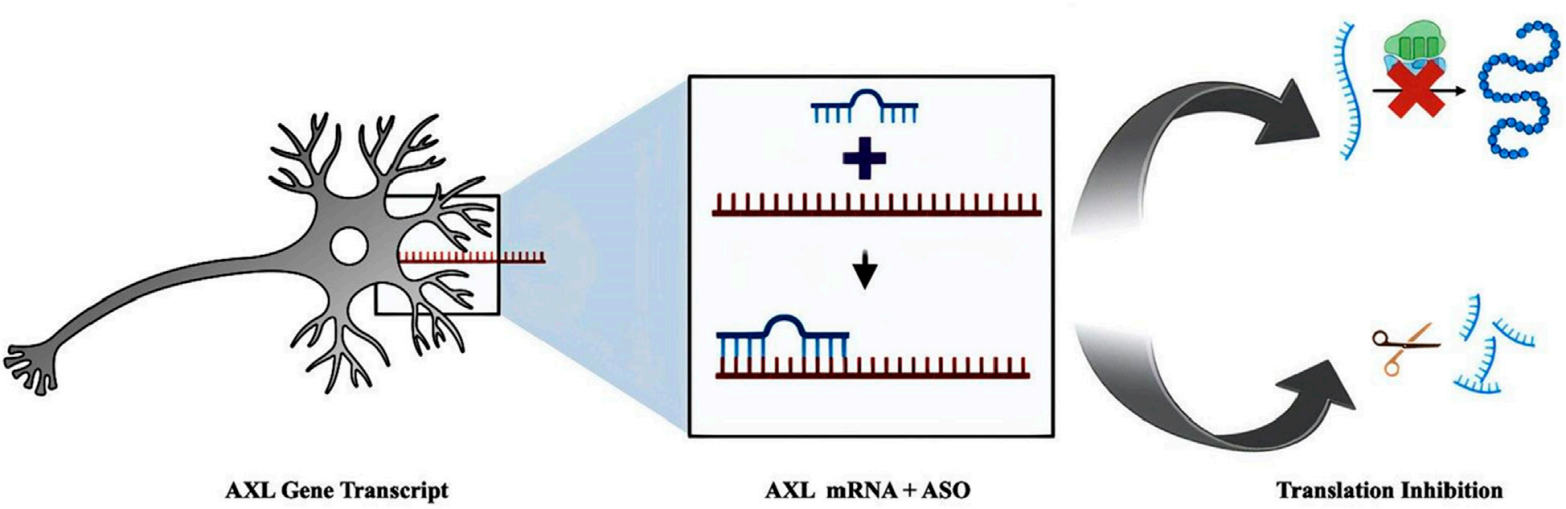
Graphical representation of ASO blocking translation of AXL Gene.

The development of growth and survival factors that prevent axonal and oligodendrocyte loss and inflammation as well as guard against these effects is a priority in the treatment of multiple sclerosis (Baecher-Allan et al., 2018). Growth-arrest specific protein 6 (Gas6) is one growth factor related to the development, survival, and suppression of oligodendrocyte immune response (Noseworthy et al., 2000; Franklin, 2002; Raine, 1997; Fontoura et al., 2006; Frohman et al., 2006). Gas6 is a secreted protein that is widely produced in the central and peripheral nervous systems by endothelial cells and neurons. It plays a variety of physiological and pathological roles, including cell proliferation, survival, and apoptosis (Torii and Yamauchi, 2016) (Figure 1).

**FIGURE 2:**
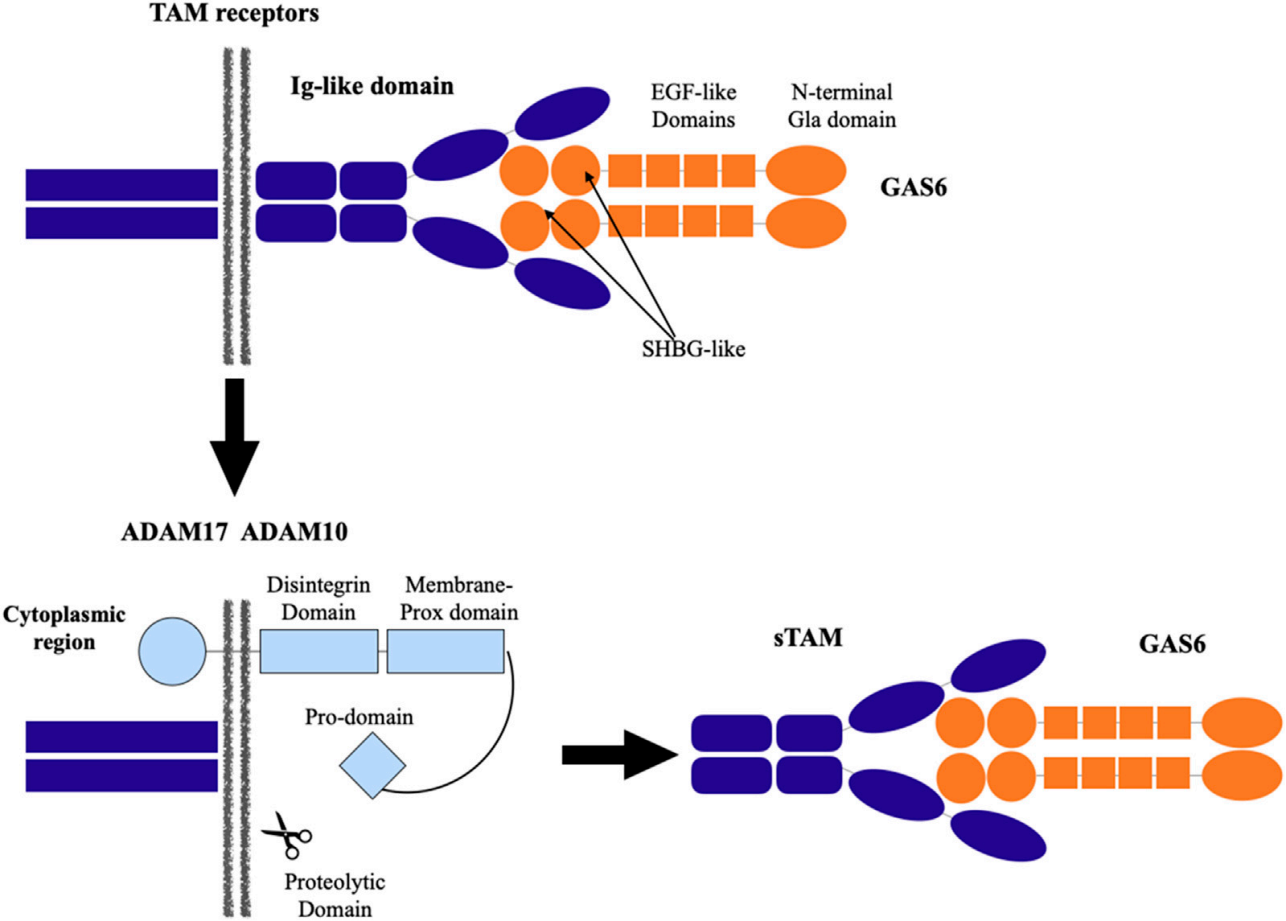
Interaction between TAM (Tyro, Axl, and Mer) receptors and growth arrest-specific receptor 6 (Gas6).

Tyro3 (Rse/Dtk/Sky), Axl (Ufo), and Mer (Eyk) are members of the TAM family of receptor tyrosine kinases, which Gas6 binds to and activates (Stitt et al., 1995). All three receptors are expressed by a wide variety of cell types, and both homophilic and heterophilic interactions can activate a receptor. The interactions between these TAM and Gas6 are shown in Figure 2 (O’Bryan et al., 1995; Thorp et al., 2011; Bellan et al., 2016). The major and minor Gas6 binding grooves are found in Axl. Tyro3 and Mer only share the minor groove, therefore the response to Gas6 is mediated in a concentration-dependent manner with a preference for Axl over Tyro3 over Mer. TAM receptor cleavage is carried out by two metalloproteinases called ADAM10 and ADAM17. In close proximity to the transmembrane region, their proteolytic domain cleaves TAM receptors, generating soluble TAM (sTAM). sTAM receptors reduce the number of ligand sites on the cell membrane and serve as decoy receptors to suppress the Gas6 function (Goruppi et al., 1999).

Over the years, various treatments and drugs have been developed to suppress the immune system and provide potential benefits such as myelin preservation and remission of symptoms. However, these therapies are not without their drawbacks, as they can lead to adverse effects (AE) ranging from mild to severe (Talanki Manjunatha et al., 2022). In the current study, a promising approach involving the use of Antisense Oligonucleotides (ASOs) targeting the mRNA of the AXL gene to mitigate adverse effects associated with MS treatment. The advantages of ASOs over small molecules are mentioned in Table 1.

**TABLE 1:** Advantages of antisense oligo therapeutics over small molecule therapeutics (Quemener et al., 2022; Yin and Rogge, 2019; Shen and Corey, 2018).

ASOs	Small molecules
High Specificity and potency	Less selective but potent
Minimal drug-drug interactions	Drug-drug interactions
Prolong duration of action	Shorter duration of action
Lower accumulation	High toxicity
IV, IVT, SC orally not available	Primarily oral
Low risk off-target activity	High risk off-target activity

We have targeted the AXL gene for the prevention of Multiple Sclerosis using ASOs (antisense oligonucleotides), which provides a rapid and high-throughput method for silencing AXL gene expression. The antisense technology relies on Watson-Crick base-pairing interactions for its specificity, and the most successful knockdowns alongside this method are frequently seen with an RNase H-dependent and RNase H-independent cleavage mechanism of the mRNA targets. ASOs targeting the 5′ and 3′ UTR Sterically blocks ASOs can also physically hinder or prevent translation or splicing (Dhuri et al., 2020; Boros et al., 2022; Baharlooi et al., 2022; Bilanges and Stokoe, 2005).

The specificity of the biological effects shown by ASOs has, however, been the subject of debate over the past several years. The oligonucleotide backbone has been modified to reduce harmful and non-specific effects, limit oligonucleotide degradation (from endogenous nucleases), and increase specificity and affinity (Scoles et al., 2019; Kulkarni et al., 2021).

## 2 Materials and methods

### 2.1 Protein-protein interaction network for AXL gene

AXL protein-protein interaction network was performed using the String database. Publication involving Multiple sclerosis data was selected for further constructing a protein-protein interaction network. The network was imported into Cytoscape for further examination. Following this, the network analyzer tool was used to obtain the expression data for all nodes in the network (Shannon et al., 2003; Szklarczyk et al., 2011).

### 2.2 Retrieval of the whole mRNA sequence of AXL gene from GenBank

The mRNA sequence of the targeted *Homo sapiens* AXL receptor tyrosine kinase (transcript variant 3 mRNA) was retrieved in FASTA format from NCBI Gene Bank with an accession no: *>NM_001278599.2*. The open reading frame was predicted using the Translate tool from Expasy.

### 2.3 Design of anti-sense oligonucleotides

ASOs were designed using the sfold (http://sfold.wadsworth.org/) web server and the ones with the highest Binding energy and minimum cross-reaction with other gene transcripts were chosen for the analysis. The optimal ASO were selected based on factors such as binding energy, GC content, and avoidance of GGGG motifs. Stronger binding is indicated by a smaller binding energy, with a desired value of −8 kcal/mol for designing potent ASOs. This threshold is based on established guidelines for antisense oligonucleotide design, where binding energies in this range have been shown to ensure strong hybridization with the target RNA, enhancing efficacy while minimizing off-target interactions. The binding energy of the ASO is determined by the weighted sum of DNA/RNA stacking energies for the hybrid formed between the ASO and the target sequence. The sfold software provides computational tools for the rational design of RNA-targeting nucleic acids such as small interfering RNAs (siRNAs), antisense oligonucleotides, and trans-cleaving ribozymes for gene knock-down investigations. The process for designing siRNAs is based on a mix of siRNA duplex thermodynamic parameters, RNA target accessibility prediction, and empirical design principles. In order to take into account, the likelihood that a population of structures for the target mRNA exists, our method for evaluating target accessibility is a novel modification of the underlying RNA folding algorithm. The application module Srna includes complete features for statistical representation of sampled structures in addition to the application modules Sirna, Soligo, and Sribo for siRNAs, antisense oligos, and ribozymes, respectively (Ding et al., 2004; Bennett et al., 2019; Crooke et al., 2018; Hendling and Barišić, 2019; Crooke et al., 2021).

### 2.4 Molecular docking of AXL-ASOs and the target mRNA

Molecular docking was performed using Schrodinger protein-protein docking PIPER. Molecular docking involves the preparation of three-dimensional structure prediction of target mRNA, Antisense oligos and Docking.

### 2.5 Three-dimensional structure prediction of target mRNA

#### 2.5.1 Secondary structure prediction using mfold

Secondary structure prediction from the sequence: the mRNA secondary structures were predicted using RNA folding form of mfold web server (http://www.unafold.org/mfold/applications/rna-folding-form.php) based on energy minimisation techniques. The sequences were selected at ionic concentrations of Na+ at 120 mM and Mg at 5 mM. So, the prediction was also done keeping the same ionic concentration. In mfold, all possible secondary structures are approximated based on Watson-Crick base pairing and the most thermodynamically stable structures are selected. The structure with minimum free energy was selected. Mfold gives the predicted results in various formats such as ct file, vienna, pdf, png, etc, (Zuker, 2003).

#### 2.5.2 ssRNA tertiary structure prediction by RNA composer

The three-dimensional structure of target RNA was predicted using RNA composer: the input for RNA composer is ct file from mfold secondary structure prediction which is converted to dot-bracket notation by the server itself and is sent to the home page for prediction of 3D structure of the RNA. This web server operates on the RNA FRABASE database which has RNA 3D structures. This server searches the fragment of the RNA input in secondary structure in dot-bracket notation and gives the 3D structure in pub format. The output is a pdb structure of the RNA the same structure was utilized for Docking (SantaLucia, 1998).

### 2.6 Three-dimensional structure prediction for antisense oligos

#### 2.6.1 Secondary structure prediction using mfold

Secondary structure prediction from the sequence: ssDNA secondary structures were predicted using the DNA folding form of mfold web server (http://unafold.rna.albany.edu/?q=mfold/DNA-Folding-Form) which uses the free energies from the laboratory of SantaLucia (1998). The sequences were selected at ionic concentration of Na+ at 120 mM and Mg at 5 mM. So, the prediction was also done keeping the same ionic concentration. In mfold, all possible secondary structures are approximated based on Watson-Crick base pairing and the most thermodynamically stable structures are selected. In addition to the predicted secondary structure of the ssDNA this step provides the minimum free energy of the fold. The structure with minimum free energy was selected. Mfold gives the predicted results in various formats such as ct file, vienna, pdf, png, etc, (Zuker, 2003).

#### 2.6.2 ssRNA tertiary structure prediction by RNA composer

ssRNA tertiary structure prediction using RNA composer: the input for RNA composer is ct file from mfold secondary structure prediction which is converted to dot-bracket notation by the server itself and is sent to home page for prediction of 3D structure of ssRNA. This server automatically takes “t or T” as ‘U’ and gives the tertiary structure of RNA. This web server operates on the RNA FRABASE database which has RNA 3D structures. This server searches the fragment of the RNA input in secondary structure in dot-bracket notation and gives the 3D structure in pub format (SantaLucia, 1998).

#### 2.6.3 Conversion of ssRNA 3D structure to ssDNA structure

Conversion of ssRNA to ssDNA 3D structure: the conversion of three-dimensional structure of ssRNA to ssDNA was performed using an in-house developed tool RNA2DNA which takes the input as.pdb files of ssRNA 3D structure and converts it to the ssDNA by editing the uracil residues of RNA to thymine residues and Deoxygenating the Ribose sugar to deoxy ribose sugar (Biesiada et al., 2016).

#### 2.6.4 Energy minimization of predicted structure

Energy minimization of the predicted structure using the UCSF chimera tool (Jeddi and Saiz, 2017).

### 2.7 Docking using schrodinger PIPER

Three-dimensional structures of both target RNA and AXL-ASO were incorporated into the Schrodinger Maestro workspace and were prepared using protein preparation workflow. The target mRNA fragment and the ASO were docked using the PIPER protein-protein docking platform. The mRNA fragment was chosen for receptor and ASO was chosen as the ligand. PIPER uses FFT-based docking approach to efficiently predict the interactions where it employs a grid-based representation of the docking search space and computes the correlation between Fourier transforms of receptor surface to estimate interaction energies. Stochastic optimization methods are used to sample conformational space, and generated docking poses are ranked based on correlation scores. Optionally, refinement steps may be included to improve accuracy. The top 30 poses were incorporated for docking analysis and the best pose was chosen based on the number of H-bonds and the binding pose.

## 3 Results

### 3.1 Protein-protein interaction network

**FIGURE 3:**
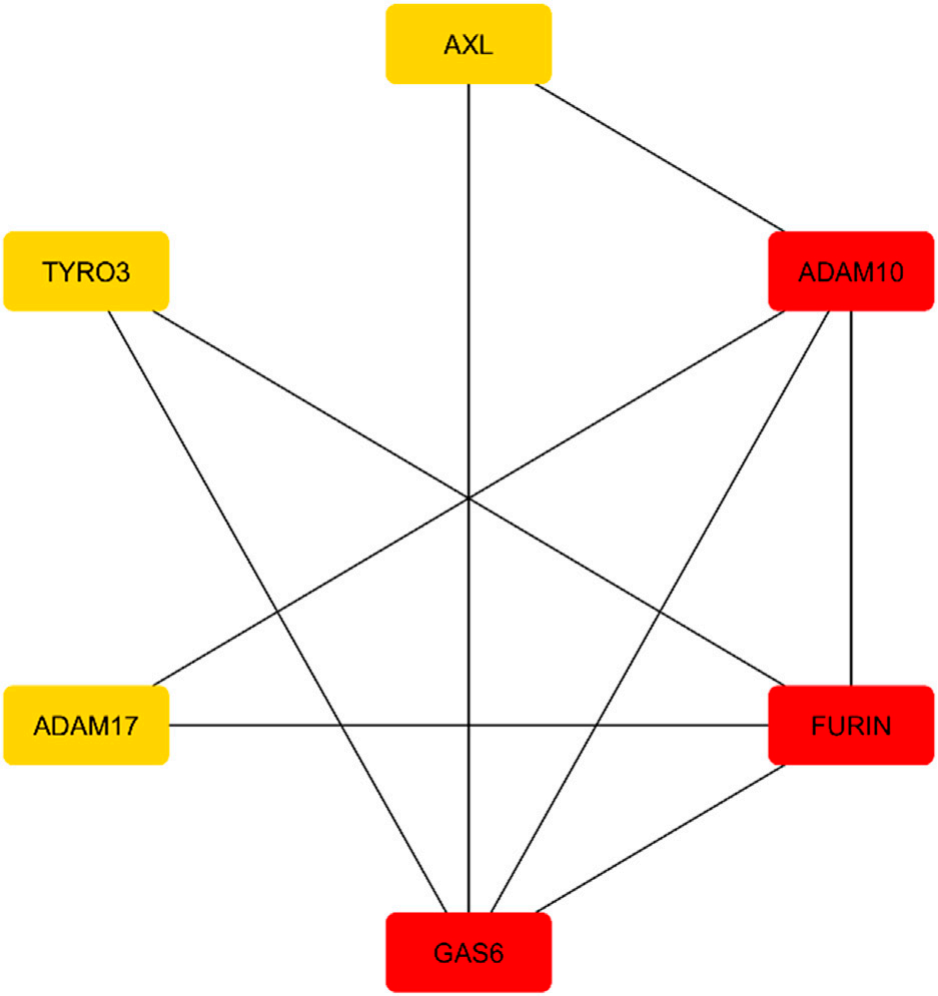
AXL protein-Protein interaction network showing ADAM10, FURIN, GAS6 as highly expressed genes.

The string database was searched for the AXL gene in the query for Pathway/Process/Disease/Publication. A publication that involves data on multiple sclerosis was chosen to aid in the development of a protein-protein interaction network. The publication involved six proteins GAS6, FURIN, ADAM10, ADAM17, TYRO3 and AXL, which formed a network consisting of a specified number of clusters. The number of nodes, edges, an average node of degree, avg. local clustering was found to be 6,9,3,0.75 respectively. The network was imported into Cytoscape for further examination. Following this, the network analyzer tool and the cytoHubba plugin were used to obtain the expression data for all nodes in the network. Top expressed nodes were colour-coded to highlight their expression degrees, facilitating further investigation and analysis. The continuous mapping option in the node-filling feature was used to identify the most highly expressed nodes based on the degree parameter. The protein networks were colour-coded according to the degree of expression. The FURIN, ADAM10, and GAS6 were highly expressed when compared to ADAM17, TYRO3 and AXL are mentioned in Figure 3.

### 3.2 Design of ASOs

The ASOs were designed to target the AXL gene transcripts using sfold server. The server gave an output of 157 ASOs. The obtained ASOs were filtered based on the criteria of GC content of 50% and above, antisense oligo binding energy above −8 kcal/mol, and minimal cross-reaction with non-target genes which was checked by performing NCBI-BLAST for the sequence similarity check and three sequences with minimum sequence similarity with non-target genes were selected as final ASOs. A1 and A2 target the 5′ UTR region of the mRNA with target position of 229–248 and 230–249 with binding energy of −9.5 kcal/mol and −10.8 kcal/mol respectively and A3 targeting the coding sequence with target position of 923–942 and Binding energy of −10.6 kcal/mol with the AXL mRNA. The final three ASOs are depicted in Table 2.

**TABLE 2:** Top ASOs targeting Axl gene for the prevention of Multiple Sclerosis.

Name	Target position on the mRNA	Target sequence	Antisense oligo	GC content	Oligo binding energy (kcal/mol)
A1	229–248	GGG​CAC​CCC​AAA​GAA​UAC​UA	TAG​TAT​TCT​TTG​GGG​TGC​CC	50.0%	−9.5
A2	230–249	GGC​ACC​CCA​AAG​AAU​ACU​AC	GTA​GTA​TTC​TTT​GGG​GTG​CC	50.0%	−10.8
A3	923–942	UCC​UAG​GCC​UUA​GAA​CCU​CC	GGA​GGT​TCT​AAG​GCC​TAG​GA	55.0%	−10.6

### 3.3 Structure prediction of ASOs

**FIGURE 4:**
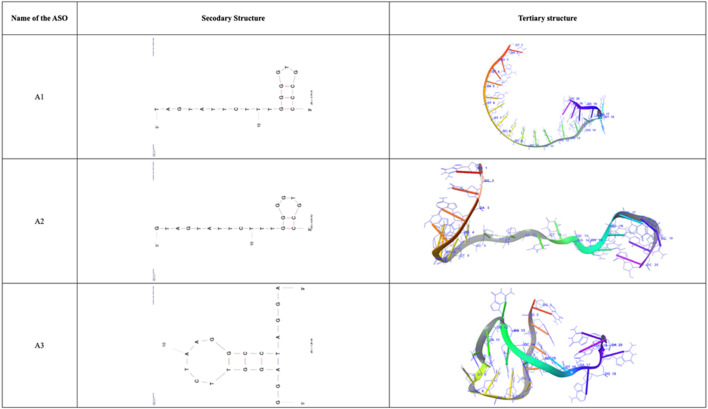
Secondary and Tertiary structures of filtered ASOs A1, A2, and A3.

The secondary structure and tertiary structure of the designed ASOs were predicted and are depicted in Figure 4. The secondary structure of the anti-AXL-ASOs had typically bulge loops, hairpin loops and single-stranded structures which are essential for binding and interaction with the target.

### 3.4 Molecular docking

**FIGURE 5:**
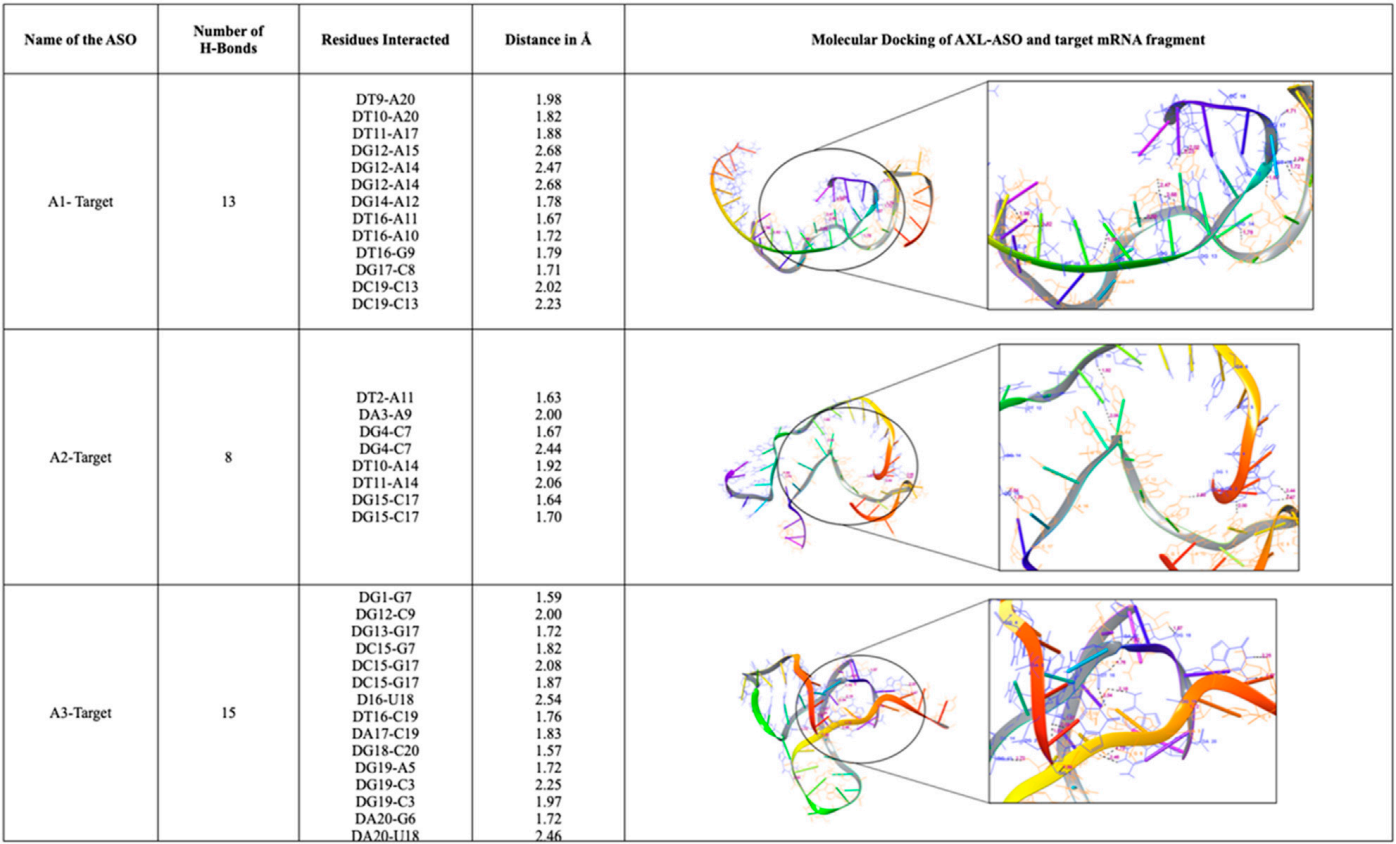
Molecular Docking studies of ASOs targeting AXL.

The three ASOs were docked to their respective target mRNA fragments. A1 was found to be forming 13 H-bonds with the corresponding target, A2 was found to be forming 8 H-bonds with the corresponding target and A3 was found to be forming 15 H-bonds with the corresponding target. The binding residues and their interactions of the respective ASOs with their targets are depicted in Figure 5.

## 4 Discussion

Multiple Sclerosis (MS) is a persistent autoimmune condition that attacks myelin and obstructs communication in the central nervous system. The TAM receptors (Tyro3, Axl, and Mer) and their ligand Gas6 play critical roles in human biology by controlling apoptosis, inflammation, and cell proliferation (Pettersen et al., 2004). In a study on multiple sclerosis by Weinger et al. (2009) found that in both chronic active and chronic silent, elevated levels of membrane-bound Mer, soluble Mer, and soluble Axl were found compared to normal tissue. While Gas6 positively correlated with soluble Axl and Mer in normal tissue, an inverse negative correlation was observed between Gas6 and soluble Axl and Mer in established multiple sclerosis lesions (Kozakov et al., 2006). Several mechanisms, including neurotrophic effects, anti-inflammatory effects on microglia, trophic effects on oligodendrocytes mediated by Axl, and pro-phagocytic activities mediated by Axl and MerTK, are probably responsible for this protection. TAM activation has a positive effect on MS lesions supported by limited but reliable evidence from human research.

The FDA approved drugs like Glatiramer Acetate, Alemtuzumab, Dimethyl Fumarate, Laquinimod, Teriflunomide, Rituximab, Daclizumab, Cladribine has shown efficacy in reducing relapse rates, functions by modulating the immune response to myelin antigens and disability progression. Adverse effects mainly include lymphopenia, increased risk of infections and other effects (Lemke, 2013). To overcome the adverse effects associated with MS treatments, a novel approach involving Antisense Oligonucleotides (ASOs) targeting the mRNA of the AXL gene has been proposed. The antisense technology relies on Watson-Crick base-pairing interactions for its specificity, and the most successful knockdown alongside this method is frequently seen with an RNase H-dependent and RNase H-independent cleavage mechanism of the mRNA targets (Wei et al., 2021).

In the current study, a protein-protein interaction network was built using string and Cytoscape tools. We specifically designed three Antisense oligos targeting AXL gene transcripts at the 5′UTR region and coding region. The sequence and target region of the ASOs with the binding affinity are given in Table 2. The secondary and tertiary structures of the ASOs were predicted. The binding affinities of the ASOs (−9.5, −10.8, and −10.6 kcal/mol) were influenced by the number and distribution of hydrogen bonds formed between the ASOs and target mRNA. For instance, A3, with the highest number of hydrogen bonds (Goruppi et al., 1999), showed the strongest interaction, indicating its potential for efficient target hybridization. Residues were critical for stabilizing these interactions, aligning with findings from previous studies that highlight the importance of hydrogen bonding in ASO-mRNA binding stability (Crooke et al., 2021; Liang et al., 2017; Lange et al., 2022). These results suggest that A1, A2, and A3 could effectively modulate AXL expression, a critical factor in neuroinflammation and oligodendrocyte dysfunction associated with MS. While the computational design and docking analyses confirm the theoretical feasibility of these ASOs, further experimental validation is necessary to evaluate their therapeutic potential. Importantly, this study lays the groundwork for exploring ASOs as a targeted approach to managing MS, complementing existing therapies that primarily focus on immune modulation. The three AXO ASOs are selected based on their binding energy to the respective target sites: A1, which has a target site between 229 and 248; A2, which has a target site between 230 and 249 binding to the 5′ UTR, and A3, which has a target site between 923 and 942, which is a coding region with an open reading frame in between the 377 and 2,257 nucleotides translating to a 626aa. ASOs targeting the 5′ and 3′ UTR Sterically block ASOs can also physically hinder or prevent translation or splicing. For instance, in human umbilical vein endothelial cells, Baker et al. (1997) created RNase H-independent 2′-O-2-methoxyethyl ASOs that were complementary to the 5′ cap region of the intercellular adhesion molecule 1 (ICAM-1) transcript. By preventing the assembly of the 80S translation initiation complex, these ASOs prevented protein production of the targeted transcript (Baharlooi et al., 2022).

This is the first approach for the treatment of Multiple Sclerosis using Antisense Oligonucleotide therapeutics targeting AXL gene. This approach aims to modulate the immune response and potentially prevent MS development with minimal adverse effects. However, further research and clinical trials are necessary to validate the efficacy and safety of this innovative strategy.

## 5 Conclusion

MS treatment options, such as Glatiramer acetate, Alemtuzumab, Dimethyl Fumarate, Teriflunomide, Laquinimod, Rituximab, Daclizumab, and Cladribine, have demonstrated varying efficacies and associated adverse effects. Although these drugs have improved the lives of many MS patients, the pursuit of safer and more effective treatments remains ongoing. The exploration of novel approaches, such as Antisense Oligonucleotides targeting the AXL gene, holds promise for minimizing adverse effects while effectively managing MS. Continued research and advancements in MS therapeutics will ultimately provide improved outcomes and better quality of life for individuals affected by this debilitating disease.

## Data Availability

The original contributions presented in the study are included in the article/supplementary material, further inquiries can be directed to the corresponding authors.

## Abbreviations

MS, multiple sclerosis; ASOs, Antisense Oligonucleotides; CNS, Central Nervous System; AE, Adverse Effects; AD, Alzheimer’s Disease; HD, Huntington’s Disease; ALS, Amyotrophic Lateral Sclerosis, ICAM-1, Intercellular Adhesion Molecule 1.
